# Developing a Clinic-Based, Vaccine-Promoting Intervention for African American Youth in Rural Alabama: Protocol for a Pilot Cluster-Randomized Controlled Implementation Science Trial

**DOI:** 10.2196/33982

**Published:** 2022-04-08

**Authors:** Henna Budhwani, Vinita Sharma, Dustin Long, Tina Simpson

**Affiliations:** 1 Department of Health Policy and Organization School of Public Health University of Alabama at Birmingham Birmingham, AL United States; 2 Department of Epidemiology College of Public Health and Health Professions and College of Medicine University of Florida Gainesville, FL United States; 3 Department of Biostatistics School of Public Health University of Alabama at Birmingham Birmingham, AL United States; 4 Department of Pediatrics School of Medicine University of Alabama at Birmingham Birmingham, AL United States

**Keywords:** human papillomavirus, COVID-19, vaccine, adolescents, rural, African American, implementation science

## Abstract

**Background:**

African American youth in rural Alabama are clinically underserved and have limited knowledge about the human papillomavirus and the novel coronavirus 2019 (COVID-19) vaccines, including knowledge about the risk for developing cervical or oropharyngeal cancers or COVID-19.

**Objective:**

In this 30-month study, we propose to develop an in-clinic, youth-tailored, vaccine-promoting intervention for vaccine hesitancy reduction that can be seamlessly integrated into the existing environments of pediatric and family practice settings in rural Alabama.

**Methods:**

This exploratory, sequential mixed methods study will be conducted in 3 phases. In the first phase, we will assess stakeholders’ knowledge, sentiments, and beliefs related to vaccination in general, COVID-19 vaccination, and human papillomavirus vaccination. We will also assess stakeholders’ perceptions of barriers to vaccination that exist in rural Alabama. This will be followed by a second phase wherein we will use the data collected in the first phase to inform the development and finalization of a noninvasive, modular, synchronous counseling intervention that targets the behaviors of 15- to 26-year-old adolescents. In the third phase, we will conduct a pilot hybrid type 1 effectiveness-implementation cluster-randomized controlled trial to assess intervention acceptability and feasibility (clinics: N=4; African American youth: N=120) while assessing a “clinical signal” of effectiveness. We will document implementation contexts to provide real-world insight and support dissemination and scale-up.

**Results:**

The study was funded at the end of December 2020. Approval from the University of Alabama at Birmingham Institutional Review Board was obtained in May 2021, and the qualitative data collection process outlined in the first phase of this project concluded in November 2021. The entire study is expected to be complete at the end of December 2023.

**Conclusions:**

The results of the trial will provide much needed information on vaccine hesitancy in rural Alabama, and if found efficacious, the intervention could notably increase rates of vaccinations in one of the most underserved parts of the United States. The results from the trial will provide information that is valuable to public health practitioners and providers in rural settings to inform their efforts in increasing vaccination rates among 15- to 26-year-old African American youth in rural southern United States.

**Trial Registration:**

ClinicalTrials.gov NCT04604743; https://clinicaltrials.gov/ct2/show/NCT04604743

**International Registered Report Identifier (IRRID):**

DERR1-10.2196/33982

## Introduction

### Background

National estimates indicate that about half (49%) of adolescents (aged 13-17 years) are up-to-date on their human papillomavirus (HPV) vaccines, with about 66% of same-aged adolescents having received at least 1 dose of the HPV vaccine [1]. Notably, fewer adolescents in rural settings accept the HPV vaccine [1]. Adolescents in rural communities have an HPV vaccination rate that is 15% lower than that of their peers in urban centers (at about 34%) [2]. In Alabama, the average HPV vaccination rate (series completion) is about 20%—nearly 30% lower than the national average and 14% lower than the average for rural communities [3]. Some Alabama rural counties have HPV vaccination rates that are as low as 9%, with many Alabama counties clustering between 10% and 25% [3]. These rural counties exhibit high rates of poverty, have limited access to health care, and are home to many African Americans. For example, Alabama’s Greene Country has an HPV vaccination rate of 18%. Further, 80% of Greene Country residents identify as African American, residents’ average household income is US $21,339, and only 16% of residents hold a college degree or higher [4].

To date, almost 1 million American lives have been lost to COVID-19 [5]. African Americans are disproportionally affected by COVID-19 morbidity and mortality. Reducing COVID-19 vaccine hesitancy in rural Alabama is a high-priority, urgently warranted public health target. Focusing on increasing HPV vaccine uptake and concurrently examining COVID-19 vaccine hesitancy are timely and warranted targets due to low rates of HPV vaccination and high levels of COVID-19 vaccine hesitancy [6], of which both are devastating African American populations in rural communities.

Research elucidating the barriers to HPV and COVID-19 vaccination in rural settings is urgently warranted. Findings from such research can be applied to developing sustainable and scalable interventions that, if acceptable and feasible, could lead to improved rates of vaccination and reduced rates of vaccine-preventable disease among African Americans living in rural America.

### Theoretical Framing

Our study is guided by the Information-Motivation-Behavioral Skills (IMB) theory, which is widely used to guide the development of prevention interventions [7]. The IMB theory was developed by considering the psychological determinants of risk and prevention behavior; IMB integrate constructs of social and health psychology theories (eg, social cognitive theory) [8-11]. IMB translate theories into potential intervention targets. Our proposed intervention will improve knowledge of HPV, the HPV vaccine, and the COVID-19 vaccine (information) and reduce stigma, distrust, and hesitancy (motivation), leading to improved vaccine confidence and higher vaccination rates (behavior skills).

### Vaccine Hesitancy

The IMB theory is particularly suited to addressing vaccine hesitancy—a complex and multifaceted phenomenon [12,13] harming individuals and broader communities. Although vaccine-focused health disparities research has indicated gaps in uptake [14-16], studies that hone in on vaccine hesitancy have noted reasons for these gaps. A recent study of adolescent perspectives in rural Alabama found significant misinformation and institutional distrust, which resulted in vaccine skepticism [6]. Additionally, since adolescents are often ambivalent or apathetic to behavior change, including seeking preventive health care [17], theoretical orientations that enhance motivation without critique or judgment have the potential to resolve barriers to vaccination. The IMB theory is designed to address information (or misinformation) while building motivation, leading to enhanced behavior skills or behavior change, such as vaccine uptake.

### Protocol Summary

The purpose of this protocol is to present the design of a pilot hybrid type 1 effectiveness-implementation cluster-randomized controlled intervention trial for improving HPV and COVID-19 vaccine acceptance by African American adolescents in rural Alabama.

## Methods

### Ethics Review and Approval

All study materials and procedures were reviewed and approved by the University of Alabama at Birmingham Institutional Review Board (approval number: IRB-300006490).

### Study Design

**Figure 1:**
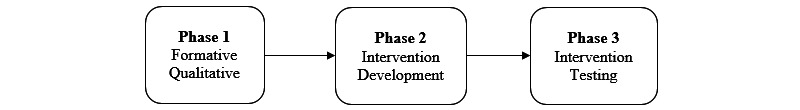
The three phases of the study.

Our study will follow an exploratory, sequential mixed methods design [18] that begins with an exploratory, qualitative phase and then moves sequentially to a single quantitative phase or multiple quantitative phases. Simultaneity is the basis for the selection of a sequential design [19]; in this sequential design, the qualitative component (phase 1) will precede the quantitative components (phases 2 and 3). The results of the qualitative phase will be used to inform the next phases [18]. The study will be conducted over the following three phases: the exploratory, qualitative phase; the intervention development phase; and the implementation and evaluation phase (Figure 1). In the third phase, we will conduct a pilot hybrid type 1 effectiveness-implementation cluster randomized controlled trial [20] to assess intervention acceptability and feasibility (clinics: N=4; adolescents: N=120).

### Recruitment

For the first and second phases of the study, participants will be recruited through our health care partner—the University of Alabama at Birmingham Selma Family Medicine clinic—and their local community–based network, which includes high schools and nonprofit agencies. During the third phase, in which the newly crafted intervention will be tested, participants will be recruited in enrolled clinics. Screening for eligibility (eg, vaccine status, age, etc) will occur via self-report and be corroborated via clinical record and immunization registry verification.

### Phase 1: Qualitative Assessment

We will conduct in-depth interviews with adolescents (age: range 15-17 years; estimated number of participants: range 12-24; number of adolescent girls: range 6-12; number of adolescent boys: range 6-12) and with the guardians of adolescents (estimated number of guardians: range 6-12). The goal of these qualitative interviews is to explore knowledge, cultural considerations, beliefs, and barriers related to vaccination to inform intervention development. We will also conduct in-depth interviews with rural health providers to document inner and outer contexts—informed by the rigorous frameworks of implementation science—of vaccine acceptance and vaccine hesitancy (estimated number of health providers: range 8-12). We will use rapid qualitative analysis [21] and thematic coding and analysis methods [22] to elucidate ways in which the intervention needs to be crafted so that it is acceptable to adolescents, parents, and their health providers in rural Alabama.

Qualitative in-depth interviews will be audio-recorded by using digital recorders, and audio files will be uploaded to an encrypted server. Files will be transcribed into Microsoft Word files by an experienced transcriptionist. We will collect these data as the interviewers, with the assistance of a trained research assistant from the target population. Coding and analysis will be performed by using rapid qualitative analysis and a thematic analysis approach, in which a priori themes and subthemes from theory and literature are supplemented with emerging themes that are “grounded” [23] in data. The NVivo (QSR International) qualitative software will be used for coding and analysis.

### Phase 2: Intervention Development

We will use intervention mapping [24] and the designing for dissemination [25] approach, which will be informed by data from the first phase, to develop the intervention. We chose the 4-step intervention mapping model to inform the development process [24]. Intervention mapping is a systematic, iterative process characterized by contextual evaluation, stakeholder feedback, and the use of theories and evidence, making it an excellent fit for implementation science trials in real-world settings [24]. Using intervention mapping will allow us to prioritize key targets while considering barriers to testing and prevention.

The tablet-based intervention will include 3 modules consisting of multiple subtopics, deliver tailored (ie, for youth and rural contexts) digital content in the clinic waiting room, include motivational interviewing [26] components that are effective in evoking behavior change while being acceptable to adolescents and parents, and include ongoing support through text reminders. The intervention will be designed to reduce health provider burden and to consider practice and environmental limitations. We anticipate drafting 2 to 3 versions of the intervention before finalizing a version for pilot-testing. Each version will be reviewed by a group of adolescents and clinical health providers (estimated number of reviewers per version: range 4-6).

### Phase 3: Intervention Evaluation

We will conduct a pilot hybrid type 1 effectiveness-implementation cluster-randomized controlled trial [20] to assess intervention acceptability and feasibility (clinics: N=4; adolescents: N=120). The cluster-randomized controlled trial design minimizes the risk of contamination among participants in different arms and is ideal when settings are similar in terms of population characteristics. These trials are frequently used when interventions are to be carried out at the level of whole groups. Implementation science hybrid designs assess intervention effectiveness in real-world settings and document barriers and facilitators to delivery and sustainability [20]. Considering that our goal is to disseminate the intervention widely, we must understand the contexts that influence success and failure, so that we can plan for them and mitigate weaknesses accordingly.

**Figure 2:**
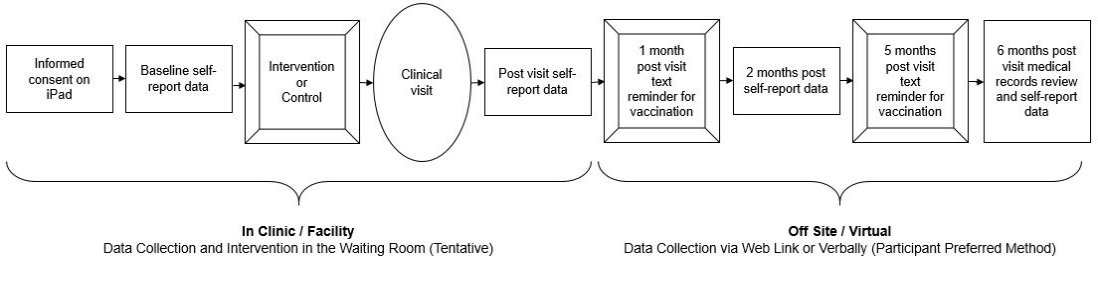
Randomized controlled trial study flow (phase 3).

We will enroll 120 adolescents across 4 clinics; 2 clinics will be randomized to implement the intervention condition, and 2 clinics will be randomized to a time-attention control. All African American patients unvaccinated for HPV (aged 15-26; all genders) will be approached to enroll in the study when they come in for a visit, regardless of the purpose of that clinical visit. After informed consent is collected from adolescents, we will collect preintervention, immediately postintervention, 2-month postintervention, and 6-month postintervention survey data related to the behavior change objectives -- increased knowledge of vaccines, reduced stigma, and increased motivation, which all result in improved vaccine confidence and higher vaccination rates — in addition to participant demographics and known potential associates of vaccination or vaccine hesitancy. We will collect design metrics to assess feasibility and acceptability, informed by the Bowen framework [27] (Figure 2).

All participant data collection will occur electronically. Informed consent will be collected via Adobe Sign (Adobe Inc), and self-report data will be collected through a standardized Qualtrics (Qualtrics International Inc) survey. Pre- and postintervention data collection, which will occur in clinics, will be conducted with iPads (Apple Inc) that are dedicated to the study site. Data will be immediately uploaded to a secure database through wireless connectivity. Self-report data to be collected at 2 and 6 months post intervention will be captured by using the same standardized Qualtrics survey that will be used in clinics; the direct survey link will be shared via text or email, depending on the participants’ preferences.

We will conduct exit interviews with clinic health providers to assess barriers and facilitators to implementation specifically to inform regional dissemination.

### Effectiveness Data

Data on clinic-level vaccination rates and vaccination series completion rates will be collected prior to the start of the study and once per year thereafter. Patient-specific data will be collected at the end of the 6-month study cycle.

### Medical Records

We propose to collect data on vaccination from medical records at partner clinics. We will request records from partner clinics, and a member of each clinic will extract them for evaluation. The research team will review medical records for evidence that participants accepted vaccination during the 6-month study period, as well as evidence of whether the participants completed the vaccine series.

### Feasibility Data (The Bowen Model [27])

Clinic staff will maintain logs on acceptances and refusals to study participation. We will use these data to assess if the study procedures and protocols are feasible and the intervention is acceptable (Table 1).

**Table 1:** Components of the Bowen model [27].

Component	Assessment focus	Sample outcomes
Demand	Documenting the use of the intervention	Fit within organizational culture and perceived effects and demand
Acceptability	Health providers’ and patients’ reactions to the intervention	Satisfaction, intent to continue use, and perceived appropriateness
Implementation	Extent to which the intervention is implemented as planned	Degree of execution and resources for implementation
Practicality	Extent to which intervention can be implemented in rural Alabama	Cost analysis factors affecting implementation
Adaptation	Modifications needed to accommodate the context	Degree to which outcomes are obtained with the mobile format
Integration	Level of system change for integrating the intervention	Perceived fit with infrastructure and perceived sustainability
Expansion	Potential success of the intervention in a different setting	Fit with the local goals and culture
Limited efficacy testing	Small-scale implementation	Effect size estimation and effects on key intermediate variables

### Implementation Data

At 4 months after the start of the trial, we will solicit feedback from staff who were interviewed in the initial qualitative phase (estimated number of staff members: range 4-8). We will collect data on the inner and outer contexts affecting implementation and data on the policies, environments, funding, and social conditions that influenced intervention delivery; this interview will take about 1 hour. We will also conduct in-depth exit interviews with a purposively selected group of adolescent participants on a rolling basis after they have concluded the 6-month study cycle (number of participants: range 6-12). The interviews will collect data on how the intervention was experienced and internalized. We will ask questions on how sociocultural nonintervention contexts affected the likelihood of vaccination and series completion. These data will provide a direct look into real-world implementation considerations affecting African American adolescents.

### Phase 3: Data Management and Quantitative Analysis

A descriptive analysis of all study feasibility and outcome variables will be summarized by using means and SDs for continuous measures and frequencies and percentages for categorical measures. Between-group differences will be compared by using standard 2-tailed cluster analyses. Further, 2-sample tests for proportions [28] will be used to compare initial vaccination acceptance, the indication of dose completion, and study dropout. An adjusted McNemar test [29] will be used to determine if the intervention group had any changes in vaccine knowledge post intervention. Similar analyses will be used in the control group, and the two groups’ changes will be examined. For any continuous measure (eg, stigma), 2-sample *t* tests [30] will be used, and appropriate assumptions will be validated. Factors related to unexpected design or study implementation issues, such as systematic enrollment issues at clinics or differential times between patients’ first visit and their follow-up resulting from issues beyond common follow-up issues, will be assessed. If it is determined that these issues require analysis adjustment, we will use generalized estimating equations to compare group differences in all measures while adjusting for these factors. Generalized estimating equations require all missing data to be missing completely at random; however, as our primary outcome relates to initial vaccination acceptance, we do not expect any missingness. Likewise, we will assume that study dropout is an indicator of incomplete dosing. Missing values for other outcomes will be examined, and if missingness at random can be assumed, multiple imputations [31] will be used.

## Results

The study was funded at the end of December 2020. Approval from the University of Alabama at Birmingham Institutional Review Board was obtained in May 2021, and the qualitative data collection process outlined in the first phase of this project concluded in November 2021. The entire study is expected to be complete at the end of December 2023.

## Discussion

### Study Implications

Based on prior research with rural African American adolescents and their families and given the low rates of vaccination in rural Alabama, developing an intervention to promote vaccination among 15- to 26-year-old youth has the potential to make a notable public health impact. A novel feature of this study is its emphasis on adolescent decision-making and autonomy. This departure from prior studies that focused on changing parental or guardian behavior makes our protocol unique, and we hope that this increases its chances of making a strong impact. Furthermore, by developing this intervention, which leverages existing infrastructure, we increase the likelihood of this intervention being scalable to similar clinic settings across similar rural communities in Alabama and extending into other neighboring states, such as Mississippi and Tennessee.

### Limitations

We note some important limitations to the study. Currently, the intervention focuses on adolescents and does not focus on parents. Because adolescents can consent for their own health care at 14 years of age in the state of Alabama, we opted to focus on them instead of their parents. During the qualitative phase, we may learn about clinic features that indicate a poor fit for such a research trial. If this occurs, as we have ample credibility with rural clinics across the state, we can recruit a replacement facility.

## Abbreviations

HPV: human papillomavirus
IMB: Information-Motivation-Behavioral Skills
